# Comprehensive molecular characterization to predict immunotherapy response in advanced biliary tract cancer: a phase II trial of pembrolizumab

**DOI:** 10.32604/or.2024.049054

**Published:** 2024-12-20

**Authors:** RYUL KIM, JOO KYUNG PARK, MINSUK KWON, MINAE AN, JUNG YONG HONG, JOON OH PARK, SUNG HEE LIM, SEUNG TAE KIM

**Affiliations:** 1 Division of Hematology-Oncology, Department of Medicine, Samsung Medical Center, Sungkyunkwan University School of Medicine, Seoul, Korea; 2 Division of Gastroenterology, Department of Medicine, Samsung Medical Center, Sungkyunkwan University School of Medicine, Seoul, Korea; 3 Department of Hematology-Oncology, Ajou University School of Medicine, Suwon-si, Gyeonggi-do, Korea; 4 Samsung Advanced Institute of Health Science and Technology, Sungkyunkwan University School of Medicine, Seoul, Korea

**Keywords:** Pembrolizumab, Whole-exome sequencing, Whole-transcriptome sequencing, Biliary tract cancer

## Abstract

**Background:**

Immune checkpoint inhibitors (ICIs) are effective in a subset of patients with metastatic solid tumors. However, the patients who would benefit most from ICIs in biliary tract cancer (BTC) are still controversial.

**Materials and methods:**

We molecularly characterized tissues and blood from 32 patients with metastatic BTC treated with the ICI pembrolizumab as second-line therapy.

**Results:**

All patients had microsatellite stable (MSS) type tumors. Three of the 32 patients achieved partial response (PR), with an objective response rate (ORR) of 9.4% (95% confidence interval [CI], 2.0–25.2) and nine showed stable disease (SD), exhibiting a disease control rate (DCR) of 37.5% (95% CI, 21.1–56.3). For the 31 patients who had access to PD-1 ligand 1 (PD-L1) combined positive score (CPS) testing (cut-off value ≥1%), the ORR was not different between those who had PD-L1-positive (PD-L1+; 1/11, 9.1%) and PDL1-(2/20, 10.0%) tumors (*p* = 1.000). The tumor mutational burden (TMB) of PD-L1+ BTC was comparable to that of PD-L1-BTC (*p* = 0.630). TMB and any exonic somatic mutations were also not predictive of pembrolizumab response. Molecular analysis of blood and tumor samples demonstrated a relatively high natural killer (NK) cell proportion in the peripheral blood before pembrolizumab treatment in patients who achieved tumor response. Moreover, the tumors of these patients presented high enrichment scores for NK cells, antitumor cytokines, and Th1 signatures, and a low enrichment score for cancer-associated fibroblasts.

**Conclusions:**

This study shows the molecular characteristics associated with the efficacy of pembrolizumab in BTC of the MSS type.

## Introduction

Biliary tract carcinomas (BTCs) are a clinically and pathologically heterogeneous group of aggressive malignancies that originate from the epithelial cells of the biliary tract, including the intrahepatic and extrahepatic bile ducts and gallbladder [1]. Despite surgery performed with potentially curative intent, relapse rates are high, with approximately 60%–70% of the patients expected to experience disease recurrence. The BILCAP trial has established adjuvant capecitabine for 6 months following radical resection as a novel standard of care [2], the role of neoadjuvant treatment combining chemotherapy, immunotherapy, and targeted therapy have provided interesting results, with these treatments having the potential to provide survival benefits in a selected group of BTC patients [3].

However, BTCs are usually diagnosed as an unresectable or metastatic disease status [4,5] with limited therapeutic options after progression following treatment with the standard first-line gemcitabine-cisplatin (GP) combination for years [6,7]. Recently, immunotherapy plus chemotherapy demonstrated improved overall survival (OS) compared to chemotherapy alone in BTC. In the TOPAZ-1 phase 3 trial (*n* = 341), durvalumab in combination with gemcitabine plus cisplatin significantly improved OS and progression-free survival (PFS) compared to placebo plus GP [8]. Another phase III KEYNOTE-966 trial (*n* = 533) showed significant improvement of OS with pembrolizumab plus GP compared to GP alone [9]. Through these pivotal studies, GP plus durvalumab or pembrolizumab has become the new first-line standard of care for patients with previously untreated unresectable or metastatic BTC [8,9].

Cancer immunotherapy is one of the biggest breakthroughs in the last decade of cancer research and has emerged as a novel pillar in the treatment of numerous otherwise chemo-resistant cancers [10]. Before chemoimmunotherapy was shown to be effective as a first-line treatment for metastatic BTC, there were studies on later-line immunotherapy. Data from patients with BTC who were treated with pembrolizumab in the phase Ib KEYNOTE-028 (NCT02054806) and the phase II KEYNOTE-158 (NCT02628067) trials was reported [11]. The objective response rate (ORR) was 6% to 13%, and some patients achieving tumor responses had durable antitumor response to pembrolizumab. However, unlike survival outcomes, the TOPAZ-1 and KEYNOTE-966 study showed the addition of immunotherapy slightly improved the objective response rate (ORR) compared to the chemotherapy alone.

Although treatment with immune checkpoint inhibitors (ICI) is promising, the population of patients with BTC who would benefit most from these agents is still controversial. Rather than using a limited, single biomarker, the usefulness of MMR, MSI, TMB, and PD-L1 should be evaluated in concert [12] to select the patients who most likely could benefit from ICIs.

Herein, we report a phase II trial of pembrolizumab as second-line therapy in patients with unresectable or metastatic BTC who progressed to first-line GP treatment. Additionally, we performed comprehensive molecular analyses, including genome sequencing, immunohistochemistry (IHC), and flow cytometry, to identify putative biomarkers for the therapeutic efficacy of pembrolizumab.

## Materials and Methods

### Study design and participants

This phase II study was designed as a prospective, single-arm, single-center, study (ClinicalTrial.gov identifier: NCT03110328). Inclusion criteria were as follows: (1) histologically or cytologically confirmed diagnosis of metastatic BTC, including intra-and extrahepatic BTC; (2) refractory to or relapsed during or after first-line therapy including gemcitabine or any platinum (GP); (3) at least 20 years old; (4) have at least one measurable lesion according to response evaluation criteria in solid tumors version 1.1 (RECIST 1.1); and (5) adequate organ function as defined by the protocol. We enrolled patients without regard to PD-1 ligand 1 (PD-L1) expression status.

Pembrolizumab 200 mg was administered as a 30-min intravenous infusion every 3 weeks until documented disease progression, unacceptable toxicity, or up to 24 months. Tumor responses were evaluated every two cycles according to the RECIST version 1.1 criteria. Toxicity profiles were evaluated according to the National Cancer Institute Common Terminology Criteria (NCI-CTC) for Adverse Events version 5.0. Additionally, we got a pretreatment tissue biopsy before initiating the study treatment to investigate potential biomarkers for all enrolled patients.

The primary goal of the study was to evaluate the objective response rate (ORR). Secondary objects were the disease control rate (DCR), progression-free survival (PFS), overall survival (OS), safety profile, and exploratory biomarker analysis. This study was approved by the Institutional Review Board of Samsung Medical Center (Seoul, Korea; IRB No. 2016-12-145), and the study was conducted in adherence to the Declaration of Helsinki and Guidelines for Good Clinical Practice. All patients provided written informed consent prior to enrolment.

### PD-L1 immunohistochemistry and microsatellite instability (MSI) status determination

The expression of PD-L1 was evaluated according to the previously defined method [13,14]. Specimen with a CPS of ≥1 was classified as PD-L1-positive. Tumor tissue microsatellite instability (MSI) status was determined as a well-established test.

### Sample preparation, whole exome, and transcriptome sequencing

The biomarker study of this study includes processes as follows, sample preparation, whole exome, and transcriptome sequencing. These methods were already defined in our previous studies [13,14]. Variant calling and filtering of whole-exome sequences and mutational signatures were also analyzed in well-established processing [15–18].

### Lymphocyte isolation and flow cytometry

Peripheral blood mononuclear cells (PBMCs) were isolated using Ficoll (GE Healthcare, Little Chalfont 17-5442-02, UK) density-gradient centrifugation and cryopreserved in freezing medium (Recovery™ cell culture freezing medium, Gibco, Waltham, MA, USA). After washing with fluorescent-activated cell sorting (FACS) staining buffer, cells were stained with fluorochrome-conjugated antibodies for 20 min at room temperature.

The fluorochrome-conjugated monoclonal antibodies used in multicolor flow cytometry are listed in Supplementary Table S1. Multicolor flow cytometry was performed using a Northern Lights flow cytometer (Cytek, Fremont, CA, USA), and the data were analyzed using FlowJo V10.6 software (Treestar, Ashland, OR, USA, Fig. S1).

### Sample size and statistical analysis

The sample size was calculated based on the hypothesis that the true RR is more than 25% with 95% power and to reject the hypothesis that the RR is less than 5% with 5% significance. A total sample size of 30 patients was planned for this study. The graph for PFS and OS was conducted by the Kaplan-Meier method using a log-rank test. The proportion of patients with pembrolizumab, who achieved complete response (CR), or partial response (PR) was defined as the ORR. All statistical tests were performed using R version 3.6.0 (http://www.r-project.org).

## Results

### Antitumor efficacy of pembrolizumab on metastatic BTC

**Figure 1:**
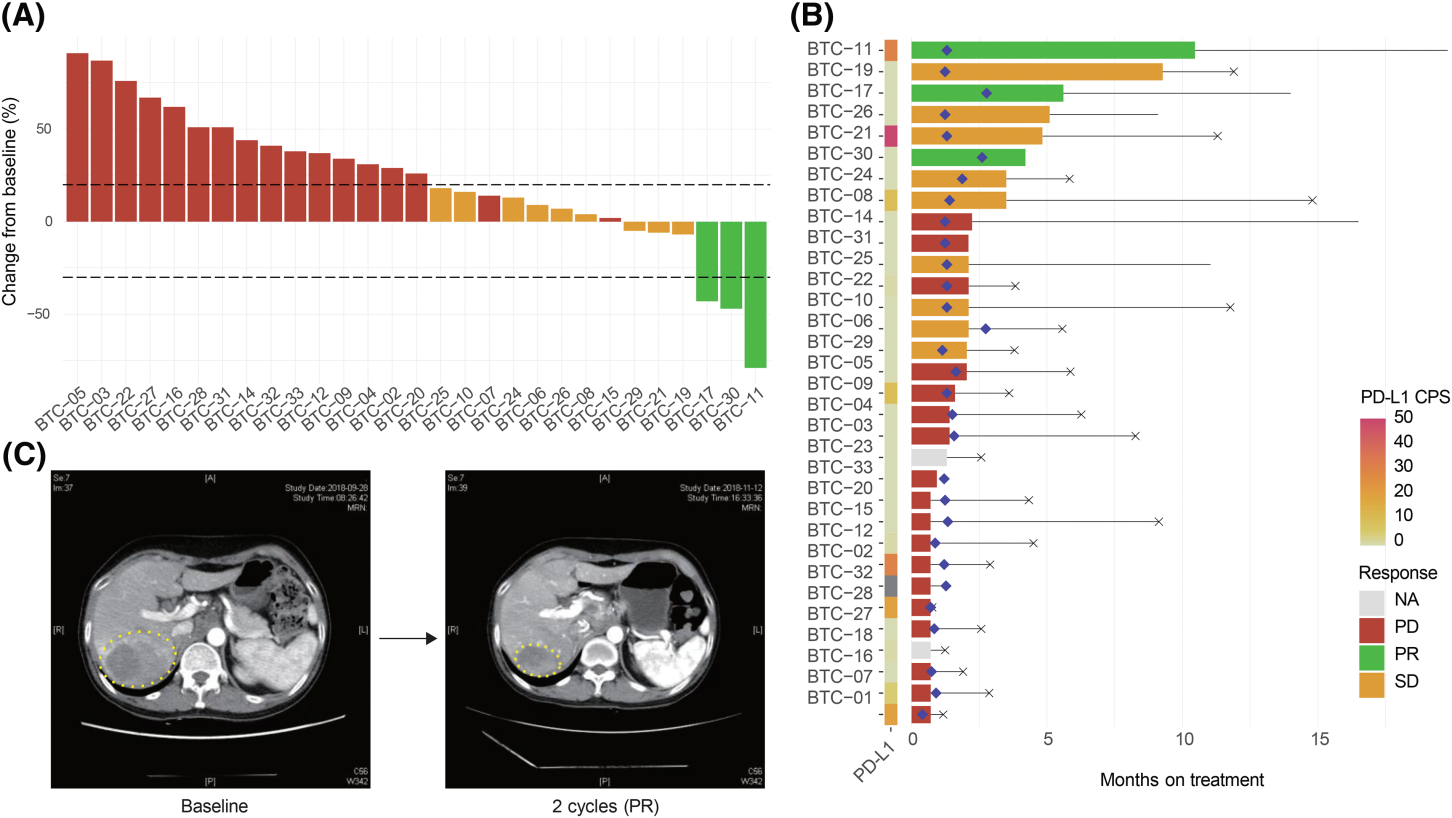
Response of chemo-refractory biliary tract cancer (BTC) patients to pembrolizumab. (A) Waterfall plot of patients to pembrolizumab. Further, 29 of 32 study participants were included in this analysis; one patient (BTC-01) rapidly developed a new metastatic lesion and two patients (BTC-18, BTC-23) were not evaluated for tumor response. (B) Swimmer plot of patients to pembrolizumab. The left panel shows a combined positive score of PD-L1 expression. Diamond-shaped points mean the time of the first response evaluation. Horizontal black lines and X-shaped points represent the duration of treatment and death, respectively. (C) Representative computed tomography images of responders (BTC-11).

Thirty-two patients with BTC who failed to respond to GP as a first-line treatment were enrolled in this study between June 2016 and February 2020 at the Samsung Medical Center (Table 1). At the data cutoff period (May 20, 2020), response evaluations were available for all enrolled participants, with a median follow-up of 4.4 (range 0.8–19.8) months. All enrolled patients had microsatellite stable (MSS) tumors. Three patients achieved PR, with an ORR of 9.4% (95% confidence interval [CI], 2.0–25.2) and nine showed SD, with a DCR of 37.5% (95% CI, 21.1–56.3; Fig. 1A). We observed PD in 18 patients (56.3%), including 8 who exhibited newly developed metastatic lesions at the time of progression (Fig. 1B). Responder was defined as the case where PR was the best response in tumor evaluation. Non-responder was defined as when a patient showed SD or PD according to RECIST v1.1. Furthermore, 11 of the 31 patients who had access to PD-L1 CPS testing (cut-off value ≥1%) had PD-L1+ tumors. The response to pembrolizumab was independent of the expression of PD-L1 and 11 (34.4%) patients showed elevated carbohydrate antigen 19-9 (CA19-9) before study treatment (Table 1). The pretreatment CA19-9 level was not different between responders and non-responders to pembrolizumab and a difference in the CA19-9 level before and after pembrolizumab was also not predictive of responses to the drug (Fig. S2). The median duration of response was 2.2 (range 0.7–10.5) months. One female patient (BTC-11, 57-year-old) received pembrolizumab as a second-line treatment following frontline GP combination and exhibited PR with a marked response after two cycles, maintaining the response for a long duration of >10 months (Fig. 1C).

**Table 1:** Distribution of patient characteristics

Characteristics	Values^#^
Age, median (range)	62 (35–77)
Sex	
Male	23 (71.8)
Female	9 (28.2)
Location	
Intrahepatic	27 (84.4)
Extrahepatic	5 (15.6)
Pathology	
Poorly differentiated	13 (40.6)
Moderately differentiated	19 (59.4)
Microsatellite instability	
Microsatellite stable	32 (100%)
Performance status	
1	29 (90.6)
2	3 (9.4)
Number of metastases	
≥2	1 (3.1)
<2	31 (96.9)
Metastasis sites	
Liver	31 (96.9)
Peritoneum	12 (37.5)
Bone	8 (25.0)
Lymph nodes	29 (90.6)
Lung	6 (18.8)
Adrenal gland	1 (3.1)
Ovary	1 (3.1)
Baseline CA19-9	
Elevated (>34 U/mL)	11 (34.4)
Not elevated	21 (65.6)

Note: ^#^Number (%) if not specified (*n* = 32).

**Figure 2:**
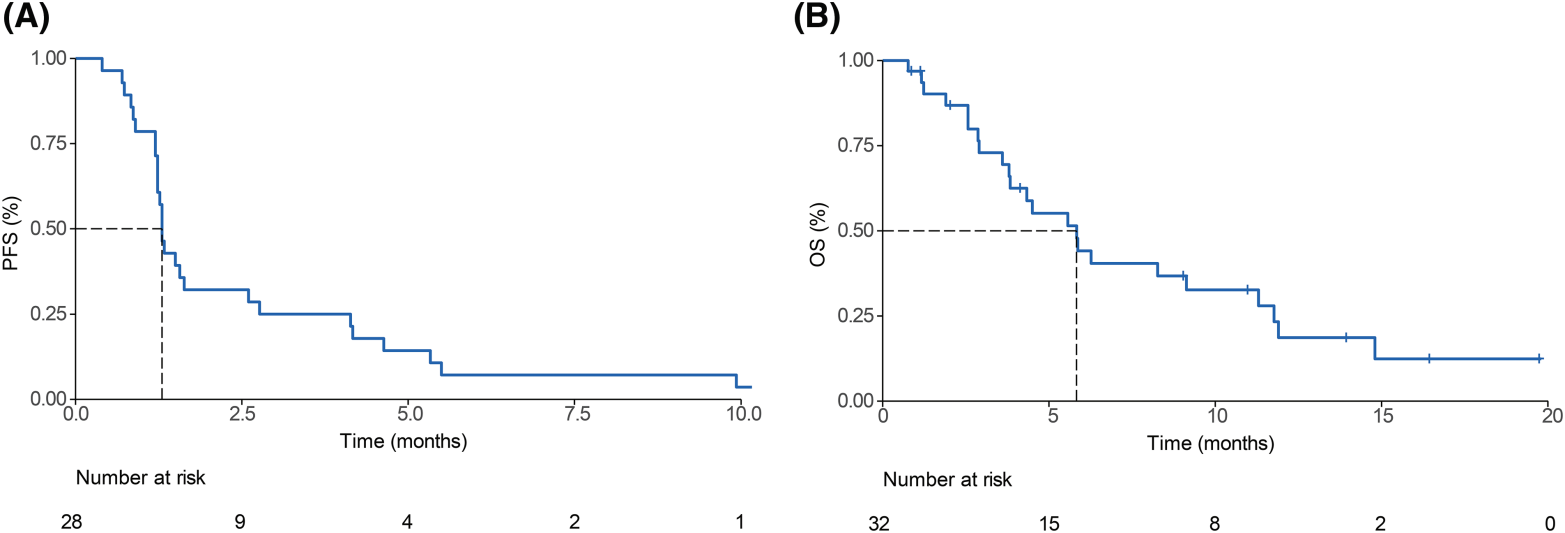
Survival outcomes of pembrolizumab-treated refractory biliary tract cancer (BTC) patients. Kaplan-Meier curves of (A) progression-free survival (PFS) and (B) overall survival (OS) among enrolled patients.

After 29 PFS events, the median PFS was 1.3 (95% CI, 1.23–2.77) months (Fig. 2A). At the data-cutoff period, 9 of the 32 patients receiving pembrolizumab had died, yielding a median OS of 5.83 (95% CI, 3.83–11.77) (Fig. 2B).

### The molecular landscape of study samples

**Figure 3:**
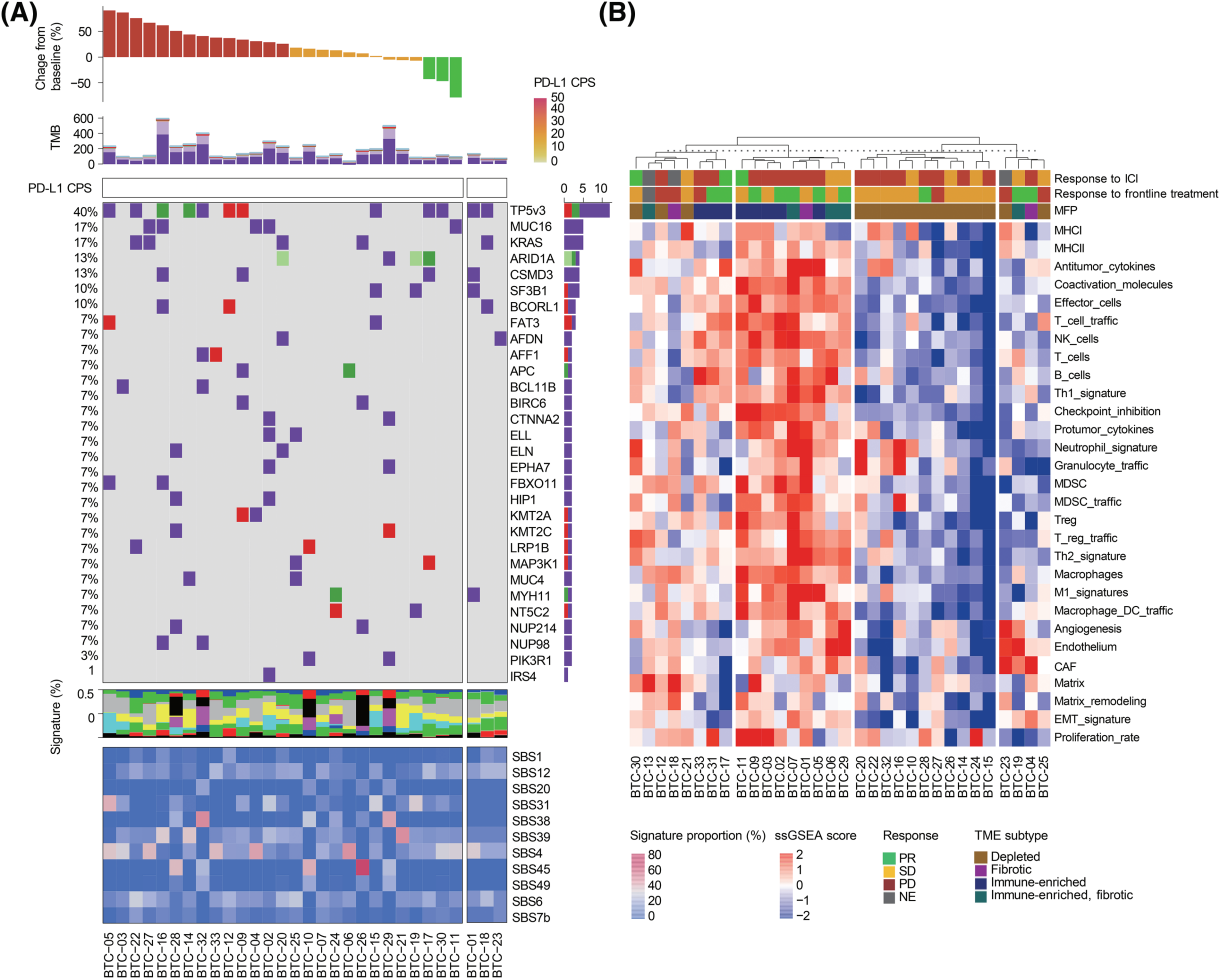
(A) Landscape of genomic alterations in patients enrolled on this study. Top to bottom: response to study treatment, non-synonymous tumor mutational burden (TMB) in the exome, combined positive score (CPS) of PD-L1 expression, oncoplot demonstrating somatic mutations, the proportion of single base substitution (SBS) subtypes in each sample, and mutational signature of somatic mutations. (B) Whole transcriptome analysis of study samples obtained from 32 BTC patients. Top to bottom: Unsupervised clustering analysis of samples, response to pembrolizumab, response to frontline treatment, tumor microenvironment (TME) subtypes, and single-sample gene set enrichment analysis (ssGSEA) scores of representative pathways.

To explore the molecular characteristics of BTC that are related to the efficacy of pembrolizumab, we analyzed the data produced from tumor sequencing. Thirty tumors with matched blood samples were included for WES. WES was conducted using a unified pipeline, resulting in a mean sequencing coverage of approximately 200x for both tumor and matched blood samples and we found high-confidence somatic mutations, including 22,258 base substitutions and 422 indels (Fig. 3A). Molecular profiles of BTCs are as heterogeneous as their pathology and biology are. The median of a variable number of somatic mutations was 133.5 (range, 39–596).

In accordance with a recent report that showed no correlation between PD-L1 expression and tumor mutational burden (TMB) in most cancer types [19], the TMB of PD-L1 + BTC was comparable to that of PD-L1-BTC (*p* = 0.630, Fig. S3). As previously reported [20], we identified the following frequent mutations: *TP53* (*n* = 12, 40.0%), *KRAS* (*n* = 5, 16.7%), and *ARID1A* (*n* = 4, 13.3%). However, no single gene was mutated in >25% of the BTC samples screened, suggesting the high genetic heterogeneity of BTC. TMB and all exonic somatic mutations were not predictive of pembrolizumab response.

We used recent pan-cancer TME signatures to examine their association with immunotherapy responses (Fig. 3B) [21]. We also classified each tumor sample into four previously defined distinct microenvironment subtypes (immune-depleted, fibrotic, immune-enriched, and immune-enriched/fibrotic) using the molecular functional portrait. Although two of the three responders had an immune-enriched TME, most samples with an immune-enriched TME were from patients who did not respond to the immunotherapy. In this analysis, the TME did not appear to affect responsiveness to immunotherapy because of the paucity of responders. Nevertheless, three responders had tumors with elevated cytokine and NK cell signatures.

### Association between peripheral immune cell profile and pembrolizumab response

**Figure 4:**
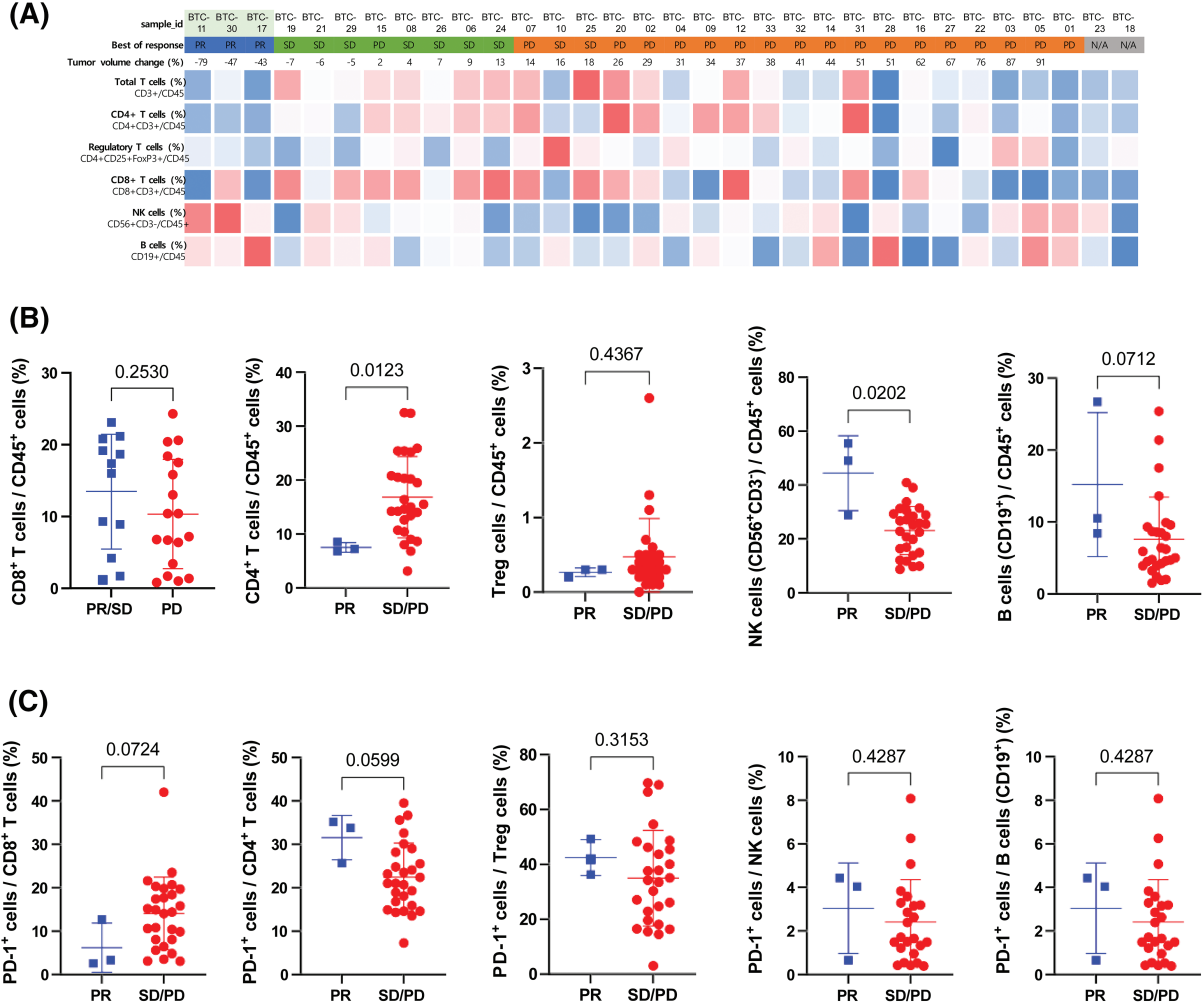
Association between immune cell profiles in peripheral blood and pembrolizumab response (A) Heatmap of the percentage of Total T (CD3+), CD4+ T (CD4+CD3+), Regulatory T (CD4+CD25+FoxP3+), CD8+ T (CD8+CD3+), NK (CD56+CD3−), and B (CD19+) cells among CD45+ cells in peripheral blood before treatment. Red and blue indicate relatively higher and lower expression levels, respectively across study patients. (B) Relative frequency of CD8+ T (CD8+CD3+), CD4+ T (CD4+CD3+), Regulatory T, NK (CD56+CD3−), and B (CD19+) cells among CD45+ cells compared between patients with partial response (PR) and stable disease (SD) or progressive disease (PD). (C) The relative frequency of PD-1+ cells among CD8+ T (CD8+CD3+), CD4+ T (CD4+CD3+), Treg (CD4+CD25+FoxP3+ ), NK (CD56+CD3−), and B (CD19+) cells was compared between patients with PR and SD or PD. Statistical analysis was performed using the Mann-Whitney test.

To investigate whether the characteristics of circulating immune cells in BTC patients are associated with the response to the drug, we analyzed pre-treatment PBMCs using multicolor flow cytometry. The proportions of T (CD3^+^), CD4^+^ T (CD3^+^CD4^+^CD8^−^), CD8^+^ T (CD3^+^CD4^−^CD8^+^), regulatory T (Treg, (CD3^+^CD4^+^CD25^+^FoxP3^+^), NK (CD3^−^CD56^+^), and B (CD19^+^) cells among the CD45^+^ cells were compared to the best response to pembrolizumab treatment in BTC patients (Fig. 4A).

There was no apparent correlation between the immune cell constitution and the best response. The proportions of CD4^+^ T, CD8+ T, Treg, and B cells among CD45^+^ cells did not demonstrate a significant difference between responders and non-responders. However, the proportion of NK cells among CD45^+^ cells was significantly higher in responders than it was in non-responders (Fig. 4B). There was no difference in the expression of PD-1 among CD4^+^ T, CD8^+^ T, Treg, and NK cells (Fig. 4C).

## Discussion

In this study, we used a comprehensive molecular approach to identify novel biomarkers that might predict the response to pembrolizumab in patients with metastatic BTC. Although previous studies were conducted to find potential biomarkers for a response to ICI monotherapy or ICI combination therapies in BTC [22–24], no significant biomarkers were identified. Comprehensive molecular analyses including whole exome/transcriptomic sequencing and blood immune cell profiling were based on tumor tissue and matched blood samples that were obtained before the initiation of pembrolizumab treatment. Thus, our data provided some insight into the molecular characteristics associated with pembrolizumab response in BTC and might be used to select a patient population that could benefit from pembrolizumab.

In this phase II trial, we comprehensively demonstrated the molecular features and efficacy of pembrolizumab as salvage therapy in BTC patients refractory to GP. PR and SD were observed in 3 and 9 patients, respectively, with an ORR of 10% and a DCR of 37.5%. Previous landmark clinical studies of ICIs have reported ORRs with pembrolizumab in advanced BTC to be 5.8% (95% CI, 2.1%–12.1%; KEYNOTE-158) and 13.0% (95% CI, 2.8%–33.6%; KEYNOTE-028). The efficacy data in our study were consistent with those reported by KEYNOTE-158 and −028. The median OS and PFS were also comparable to those reported in the KEYNOTE-028 study (median OS and PFS were 5.7 and 1.8 months, respectively) (PMID: 32359091).

Although some reported that TMB was a potential predictive biomarker for response to ICIs in BTC patients [25] and pretreatment PD-L1 expression on the tumor was significantly associated with prolonged PFS with nivolumab [26], the antitumor activity of pembrolizumab was found to be independent of PD-L1 expression and TMB in this study. Exploratory analysis of WES and the sequences revealed the heterogeneous and distinct molecular features of BTC. We identified signatures of cytokines and NK cells in tumor samples and the proportion of NK cells in peripheral blood mononuclear cells as potential biomarkers for predicting the response to pembrolizumab in BTC. Notably, both tumor and blood samples and NK cell-associated biomarkers are likely to predict the efficacy of pembrolizumab in refractory BTC. There is growing interest in circulating immune biomarkers to predict ICI efficacy in various tumors. Recently, a specific baseline immune cell population in peripheral blood including NK cells was reported to be a strong predictive biomarker of ICI treatment in NSCLC [27,28].

To determine the subgroups of BTC patients that would benefit most from immunotherapy, the identification of biomarkers predictive of response would be critical. This study was a single-arm phase II trial consisting of small sample size and responders to treatment owing to the refractory characteristics of BTC. Furthermore, it is generally very difficult to re-obtain tumor tissues available for sequencing in patients with pretreated BTC. These were obstacles to the investigation of novel markers through comprehensive molecular analysis of tumor and blood samples in BTC. Nevertheless, we successfully performed a comprehensive molecular characterization of BTC patients treated with pembrolizumab as a salvage therapy. Flow cytometry analysis of peripheral blood samples revealed that patients who showed PR had a higher proportion of NK cells than those who showed SD or PD. Although the functional impairment of PD-1-expressing NK cells has been reported in solid cancers [29,30], the molecular mechanism of PD-1 in NK cells remains poorly understood. The proportion of PD-1^+^ cells among NK cells in peripheral blood has been reported to be <10% [31]. Consequently, the predictive role of PD-1^+^ NK cells in patients who received PD-L1/PD-1 inhibitors should be interpreted with caution. However, Poznanski et al. reported that expanded NK cells remodeled the immunosuppressive TME by increasing interferon-γ production and reinforced anti-PD-1-induced reinvigoration of tumor-infiltrating T lymphocytes through a contact-independent mechanism [32]. In lung cancer patients, the association with blood NK cell activity through interferon-γ measurement and PD-1/PD-L1 inhibitor efficacy could be explained by induction of NK antibody-dependent cell cytotoxicity [33,34].

In our analysis, patients who achieved PR as the best response (BTC-11, 17, and 30) demonstrated a relatively high NK cell proportion in the peripheral blood before pembrolizumab treatment. Moreover, there was a high enrichment score of NK cells, antitumor cytokines, and Th1 signature and a low enrichment score of cancer-associated fibroblasts, which is a physical barrier and source of immunosuppressive molecules in the tissue transcriptomic data of the responders. These findings support the hypothesis that NK cells and anti-PD-L1 agents might have a synergic effect that changes the immunosuppressive TME. In line with the growing importance of NK cells for immunotherapy, a recent early-phase clinical trial showed the combination therapy with allogeneic natural killer (NK) cells and pembrolizumab exerted antitumor activity with improved efficacy compared to the recent monotherapy with pembrolizumab in patients with advanced BTC [35].

Our study has several limitations. First, our study included a relatively small number of patients; and only three patients were responders, thus, drawing definite conclusions about molecular biomarkers regarding the pembrolizumab efficacy is difficult. Second, the results of this study may be outdated because the combination treatment of chemotherapy and ICI has become the standard treatment in the first-line setting rather than the role of salvage ICI in BTC patients. Third, we intended to analyze too many variables for our relatively small sample size, which prevented us from adjusting for important factors that might have affected our results.

There continues to be an unmet need to comprehensively characterize BTC at the in-depth molecular level to further develop targeted therapies to actionable genetic abnormalities. Furthermore, molecular profiling will play an important role in the future in determining the specific prognosis of individual patients. Studies are needed to elucidate the deeper layers of biomarkers in immunotherapy in combination with chemotherapy in advanced BTC.

## Conclusion

Taken together, the findings of this phase II study demonstrated that pembrolizumab had antitumor activity in advanced BTC with MSS, which was independent of PD-L1 expression or TMB. Finally, our findings strongly suggest that NK cell-associated features in both blood and tissue samples could be potential biomarkers for pembrolizumab, which is worth further investigation.

## Supplementary Materials

Figure S1 Gating strategy of multicolor flow cytometry.

Figure S2 Baseline and 6-week follow-up carbohydrate antigen 19-9 (CA19-9) levels of responders and non-responders.

Figure S3 Tumor mutational burden (TMB) of programmed cell death 1 ligand 1 negative (PD-L1^-^) and (PD-L1^+^ biliary tract cancer (BTC).

## Data Availability

The whole data presented in this study are available upon request from the corresponding author.

## Supplementary Materials

The supplementary material for this article is available online at https://doi.org/10.32604/or.2024.049054.
