# Supplementary figures and images for: Comprehensive molecular characterization to predict immunotherapy response in advanced biliary tract cancer: a phase II trial of pembrolizumab
Source: Oncol Res. 2024 Dec 20;33(1):57–65. doi: 10.32604/or.2024.049054 (PMC11671410; doi:10.32604/or.2024.049054)

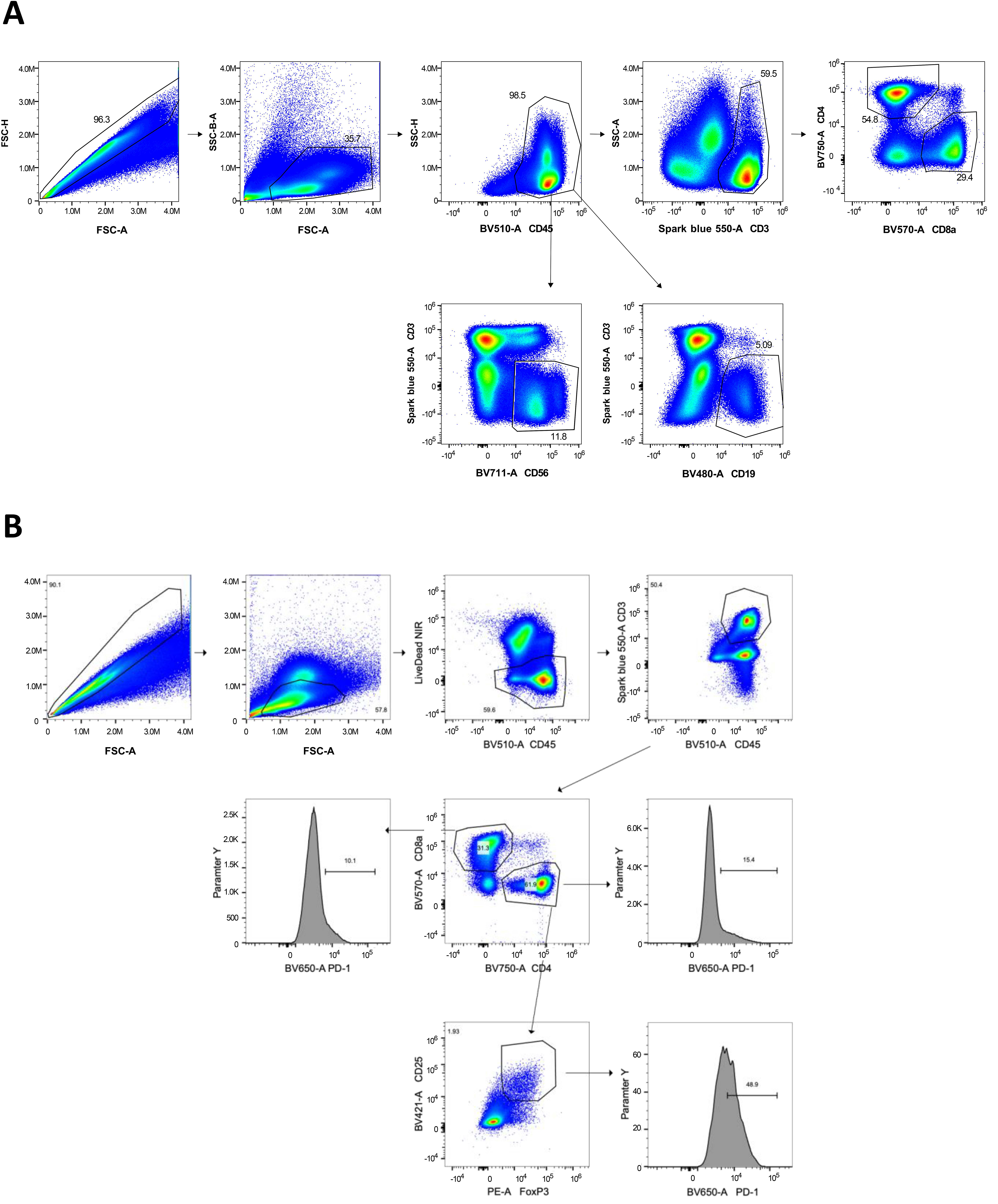

Supplement: Figure S1 [file OncolRes-33-49054-s001.tif]

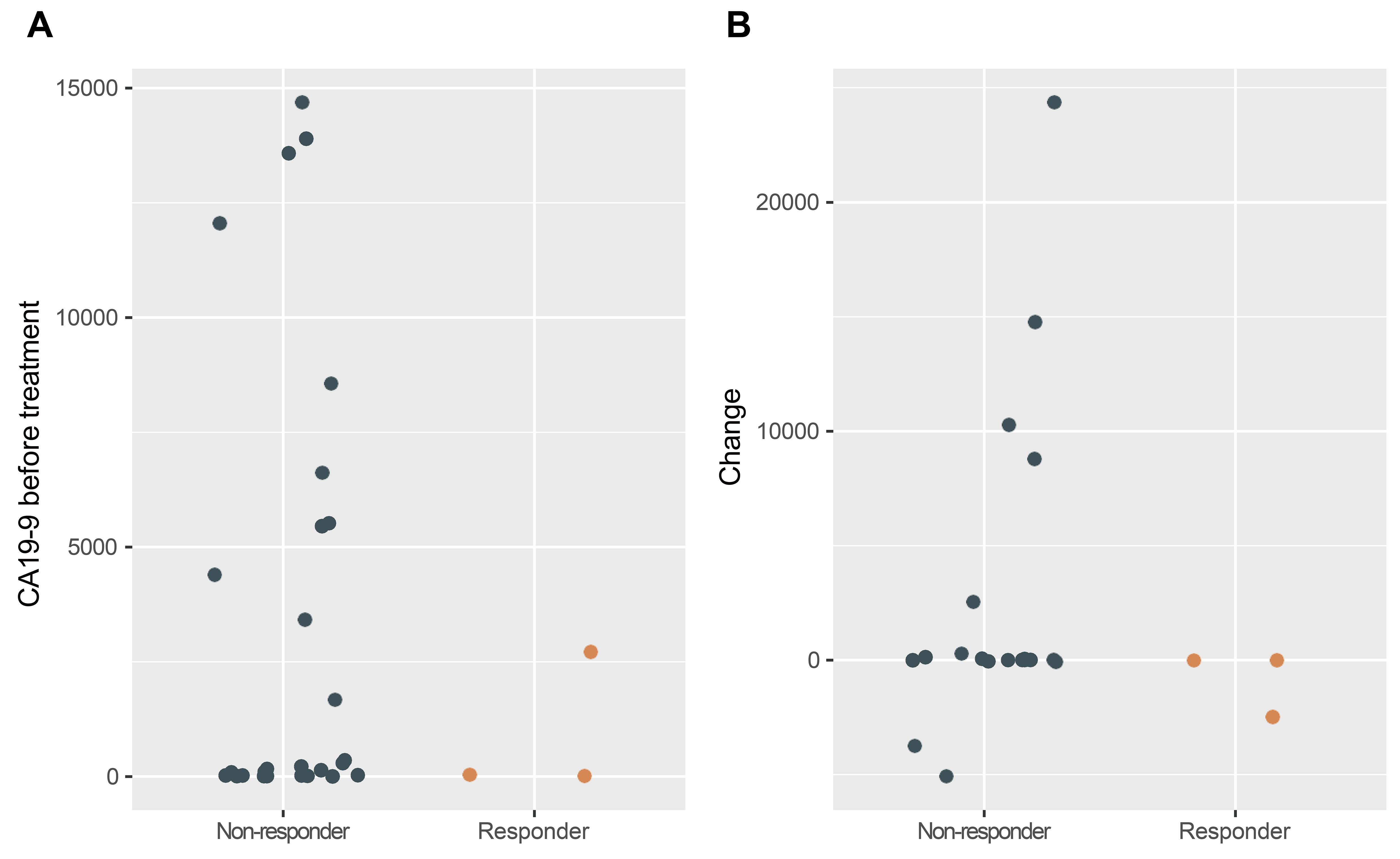

Supplement: Figure S2 [file OncolRes-33-49054-s002.tif]

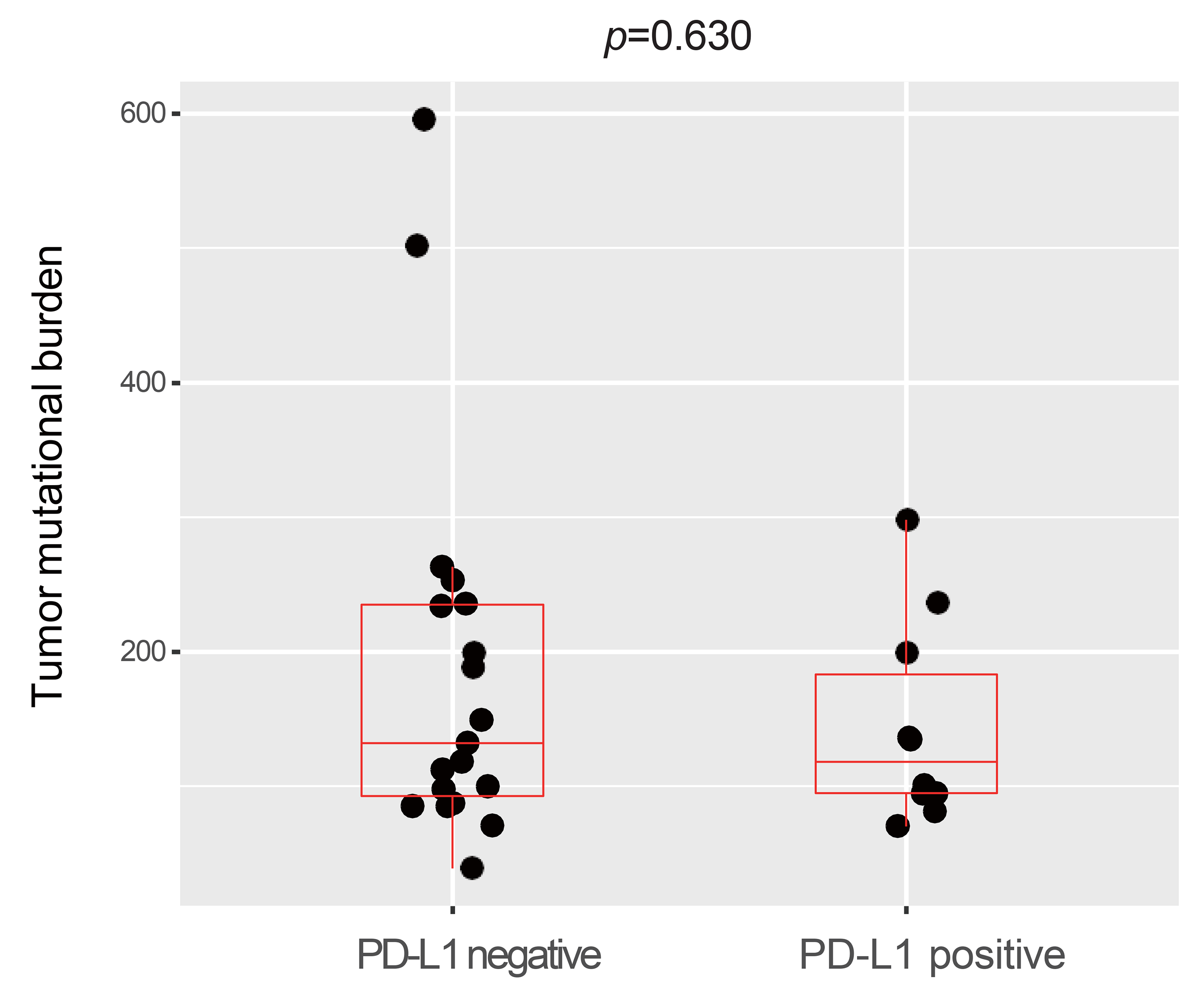

Supplement: Figure S3 [file OncolRes-33-49054-s003.tif]
