# Supplementary material for: Comprehensive molecular characterization to predict immunotherapy response in advanced biliary tract cancer: a phase II trial of pembrolizumab
Source: Oncol Res. 2024 Dec 20;33(1):57–65. doi: 10.32604/or.2024.049054 (PMC11671410; doi:10.32604/or.2024.049054)
Supplement: Supplementary file 4 [file OncolRes-33-49054-s004.docx]

**Supplementary Table S1. List of antibodies used for multicolor flow cytometry**

| **Antibody** | **Manufacture** | **Clone** | **Conjugate** | **Cat. No.** |
| --- | --- | --- | --- | --- |
| Live/Dead fixable near-IR dead cell stain | Thermo Fisher | n/a | n/a | L34976 |
| mouse anti-human CD25 | BD bioscience | M-A251 | BV421 | 562442 |
| mouse anti-human CD19 | BD bioscience | SJ25C1 | BV480 | 566103 |
| mouse anti-human CD45 | BioLegend | 2D1 | BV510 | 368526 |
| mouse anti-human CD4 | BioLegend | RPA-T4 | BV570 | 300534 |
| mouse anti-human PD-1 | BioLegend | EH12.2H7 | BV650 | 329950 |
| mouse anti-human CD56 | BioLegend | HCD56 | BV711 | 318336 |
| mouse anti-human CD8 | BioLegend | RPA-T8 | BV750 | 344846 |
| mouse anti-human CD3 | BioLegend |  | SB550 |  |
| mouse anti-human FOXP3 | Thermo Fisher | PCH101 | PE | 12-4776-42 |
